# Shade-Tolerant Soybean Reduces Yield Loss by Regulating Its Canopy Structure and Stem Characteristics in the Maize–Soybean Strip Intercropping System

**DOI:** 10.3389/fpls.2022.848893

**Published:** 2022-03-16

**Authors:** Bin Cheng, Li Wang, Ranjin Liu, Weibing Wang, Renwei Yu, Tao Zhou, Irshan Ahmad, Ali Raza, Shengjun Jiang, Mei Xu, Chunyan Liu, Liang Yu, Wenyan Wang, Shuzhong Jing, Weiguo Liu, Wenyu Yang

**Affiliations:** ^1^College of Agronomy, Sichuan Agricultural University, Chengdu, China; ^2^Sichuan Engineering Research Center for Crop Strip Intercropping System, Chengdu, China; ^3^Chengdu Da Mei Seeds Co., Ltd., Chengdu, China; ^4^Key Laboratory of Crop Ecophysiology and Farming System in Southwest, Ministry of Agriculture, Chengdu, China; ^5^Crop Research Institute, Sichuan Academy of Agricultural Sciences, Chengdu, China; ^6^Quxian Agricultural and Rural Bureau, Dazhou, China; ^7^State Key Laboratory of Southwestern Chinese Medicine Resources, Chengdu University of Traditional Chinese Medicine, Chengdu, China; ^8^CAS Key Laboratory of Mountain Ecological Restoration and Bioresource Utilization, Ecological Restoration Biodiversity Conservation Key Laboratory of Sichuan Province, Chengdu Institute of Biology, Chinese Academy of Sciences, Chengdu, China; ^9^Chuanshanqu Agricultural and Rural Bureau, Suining, China

**Keywords:** canopy structure, intercropping, lodging, soybean, photosynthesis

## Abstract

The shading of maize is an important factor, which leads to lodging and yield loss of soybean in the maize–soybean strip intercropping system, especially in areas with low solar radiation. This study was designed to explore how shade-tolerant soybean reduces yield loss by regulating its canopy structure and stem characteristics in the maize–soybean strip intercropping system. The soybean cultivars Tianlong No.1 (TL-1, representative of shade-tolerant plants) and Chuandou-16 (CD-16, representative of shade-intolerant plants) were grown in monocropping and intercropping systems from 2020 to 2021 in Chongzhou, Sichuan, China. Regardless of shade-intolerant or shade-tolerant soybean, the canopy and stem of soybean in strip intercropping were weaker than those of the corresponding monoculture. But compared with shade-intolerant soybean, the shade-tolerant soybean slightly changed its spatial structure of canopy and stem morphology and physiology in maize–soybean strip intercropping system, especially in the later growth stages. On the one hand, the canopy of shade-tolerant soybean showed relatively high transmission coefficient (TC) and relatively low leaf area index (LAI) and mean leaf angle (MLA). On the other hand, the stem of shade-tolerant soybean was obviously stronger than that of shade-intolerant soybean in terms of external morphology, internal structure, and physiological characteristics. Additionally, compared with shade-intolerant soybean, shade-tolerant soybean showed higher APnWP (the average net photosynthetic rate of the whole plant) and seed yield in the strip intercropping. The results showed that shade-tolerant soybean increased light energy capture and photosynthesis in the different canopy levels to promote the morphological and physiological development of the stem and ultimately reduce the yield loss of the strip intercropping system. However, the molecular mechanism of low radiation regulating soybean canopy structure (LAI, TC, and MLA) needs further in-depth research to provide theoretical guidance for cultivating plants with ideal canopy shape that can adapt to changing light environment in intercropping system.

## Introduction

Intercropping of maize (*Zea mays* L.) and soybean (*Glycine max* L.) has been practiced on a large scale in the world, especially in China (Du et al., 2018; Iqbal et al., 2019). This planting pattern can harvest an extra-season of soybean seeds without reducing the yield or slightly reducing the yield of maize (Hussain et al., 2020a). However, soybean plants suffer severe shading stress from maize during the intergrowth in maize–soybean intercropping systems (Zhou et al., 2019a,b; Hussain et al., 2020b), which reduces the red-to-far-red ratio of light inside the soybean canopy (Yang et al., 2014, 2020; Fan et al., 2018). These plants in this shaded environment show strong shade avoidance responses, which include reduced stem thickness, longer stem length, and petiole length and also lower photosynthetic capacity and biomass (Yang et al., 2014; Liu et al., 2016; Fan et al., 2018). In the maize–soybean strip intercropping system, similar shade avoidance responses occur in the middle and later stages of growth, which results in lodging and yield loss of soybean (Chen et al., 2020). Fortunately, the phenotypes and physiology of soybean cultivars with a different shade tolerance have corresponding shade-avoiding strategies and plasticity under shade stress.

Light is one of the essential abiotic factors for crop growth, and its intensity and quality (spectral composition) can regulate the spatial structure of canopy and photosynthesis of leaves (Huber et al., 2021; Paradiso and Proietti, 2021). In the low radiation or shading environment, the net photosynthetic rate (Pn) of soybean decreases due to the reduction in light energy captured by the soybean canopy (Yang et al., 2020), which results in the decrease of photosynthate in the whole plant (Liu et al., 2006; Fan et al., 2018). However, because of the different overlapping degrees of leaves at different canopy levels, the photosynthetic capacity of leaves at the corresponding canopy levels is also different (Jiang et al., 1993). Compared with the leaves inside the canopy, those leaves exposed to the outside can receive more light radiation (Klich, 2000), which results in an increase in Pn of leaves (Zhou et al., 2019a,b). In addition, the Pn of soybean leaves decreases with the increase in self-shading degrees, among which the Pn of the lower leaves decreases faster, which results in the accelerated aging and shedding of the lower leaves and consequently yield loss (Miyaji and Tagawa, 1979). However, the soybean cultivars differ significantly in Pn in different environments (Dornhoff and Shibles, 1970), especially in a shady environment (Hussain et al., 2020b).

The canopy spatial structure of crops can be quantitatively visualized by leaf area index (LAI), transmission coefficient (TC) and mean leaf angle (MLA). The LAI, as an important indicator of crop development, can be used to show different canopy structures (Kross et al., 2015) and plant foliage density (Walthall et al., 2004). The TC is closely related to the content and composition of pigments in the crop canopy, which can be used to comprehend spatial and temporal dynamics of photosynthetically active radiation (PAR), photosynthesis, and vegetation productivity (Gitelson et al., 2019). Increasing the MLA in the upper canopy can increase the interception of PAR (IPAR), which improves lodging resistance of stalk while increasing grain yield of maize (Xue et al., 2021). In addition, soybean cultivars with a different shade tolerance show different shade avoidance responses in intercropping systems, especially in canopy spatial structure (Gong et al., 2015). Based on the above understanding, how to improve the canopy structure of intercropping soybean to intercept more light energy has become the focus of many scholars (Keating and Carberry, 1993; Feng et al., 2018, 2019; Ren et al., 2021). Consequently, in the maize–soybean strip intercropping system, what are relationships between the spatial structure of soybean canopy and the establishment of plant morphological structure and the formation of seed yield?

Regardless of the aggravation of shade degree or long-term exposure to shade, plant is prone to lodging under the external forces such as wind (Liu et al., 2019; Hussain et al., 2020a). The phenotype and physiology of stems change when plants are subjected to shade stress, such as slender stems and lower content of structural carbohydrates (Liu et al., 2019; Hussain et al., 2021). Some studies showed that the stem strength of soybean was negatively correlated with lodging, but positively correlated with lignin content (LC) and the activity of phenylalanine ammonia lyase (PAL) and 4-coumaric acid: CoA-ligase (4CL) (Cheng et al., 2020). Cotton (Chen et al., 2014) and soybean (Liu et al., 2019) had lower LC in stems, which results in a decrease in lodging resistance index (LRI) of plants, which in turn increased lodging risk. In addition, the characteristics of stem anatomical structure are closely related to the LRI of plant stems (Hussain et al., 2021). Moreover, intercropping with low-light environment makes soybean stem slender, and the areas of xylem, phloem, and pith decrease, which further leads to the decrease of stem strength (Liu et al., 2019). However, the characteristics of stem anatomical structure are different in different soybean cultivars (Yao et al., 2018). In short, high-strength stem can reduce the lodging risk and yield loss of soybean in low-light environment.

Soybean morphology changes significantly in shading environment, which is used to describe by shading-tolerance coefficient (STC) (Li et al., 2014). The plant height (PH), stem diameter (SD), seed yield, branch, and canopy structure can be regarded as the target trait of soybean (Chen et al., 2003). Moreover, the comprehensive shading-tolerance coefficient (CSTC) is used to carefully and objectively evaluate the shade tolerance of soybean in intercropping systems (Li et al., 2014).

In this study, Tianlong No. 1 (TL-1) with high yield and Chuandou-16 (CD-16) with low yield represent shade-tolerant and shade-intolerant soybean cultivars, respectively, which had been proved by the previous research (Zhao et al., 2014; Cheng et al., 2020). The suitable density (20 plants/m^2^) with higher yield was selected for this study, according to the previous research (Cheng et al., 2020, 2021). Additionally, the strip intercropped soybean in this study was shaded by maize, which results in lodging after 49 days of sowing (V5 stage), which was proved by early study (Cheng et al., 2020, 2021). The objectives of this study are to explore how shade-tolerant soybean reduces yield loss by regulating its spatial structure of canopy and stem characteristics in the maize–soybean strip intercropping system. The results can provide theoretical reference for phenotypic modeling and soybean breeding research in maize–soybean intercropping systems.

## Materials and Methods

### Site Description

**FIGURE 1:**
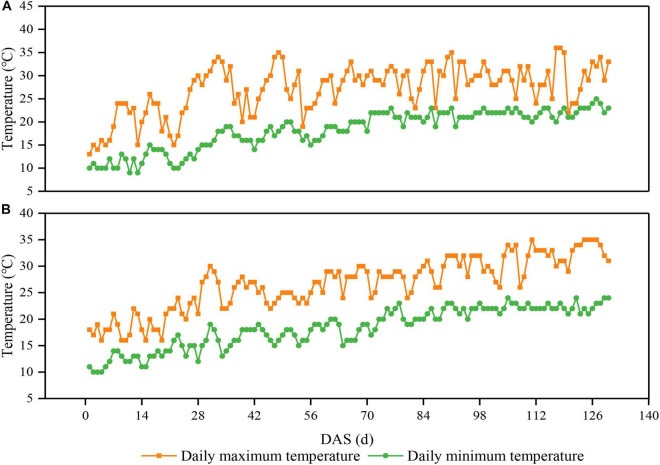
Daily maximum and minimum temperatures above the soybean canopy from sowing to harvest in 2020 **(A)** and 2021 **(B)** in Chongzhou, Sichuan, China. Orange lines and boxes represent the daily maximum temperature, whereas green lines and circles represent the daily minimum temperature. DAS represents the number of days after sowing.

The experiment was conducted from 2020 to 2021 at Chongzhou experimental site of Sichuan Agricultural University, Sichuan, China (30°33^′^N, 103°39^′^E). The daily average maximum and minimum temperature from sowing to harvest were 27.0 and 19.0°C in 2020, respectively (Figure 1A). The daily average maximum and minimum temperature from sowing to harvest were 26.8 and 17.8°C in 2021, respectively (Figure 1B). The annual sunshine and precipitation were 1,161.5 h and 1,012.4 mm, respectively. The field soil was a light loam with pH 7.1, 24.3 g/kg organic matter, 1.6 g/kg total N, 1.3 g/kg total P, 15.2 g/kg total K, 299.5 mg/kg available-P, and 169.4 mg/kg available-K.

### Experimental Design

**FIGURE 2:**
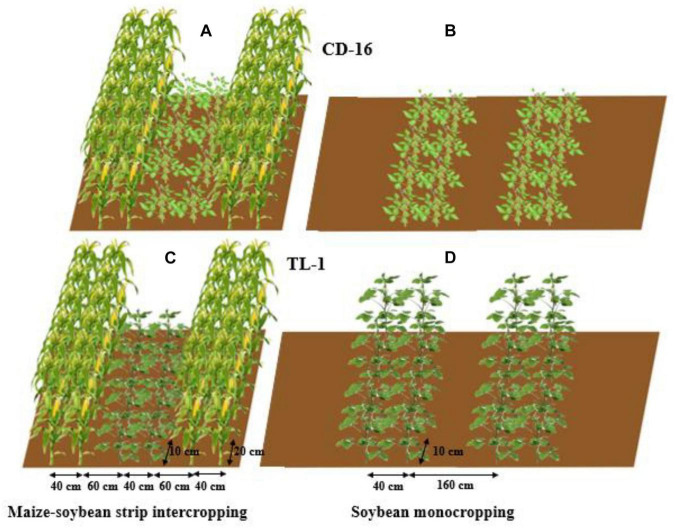
The patterns of maize–soybean strip intercropping system **(A,C)** and monoculture **(B,D)** in 2020 and 2021. Regardless of Chuandou-16 (CD-16) **(A,B)** or Tianlong NO. 1 (TL-1) **(C,D)**, the soybean plant spacing is 10 cm and the row spacing is 40 cm. The maize plant spacing is 20 cm and the row spacing is 40 cm. In monoculture, the row spacing between each soybean strip is 160 cm.

The field experiment was conducted using the two-factor randomized block design, with planting patterns (maize–soybean strip intercropping and monocropping) as the main factor whereas soybean cultivars as the secondary factor. The variety of maize was zhenghong-505 (ZH-505, semicompact plant) and soybean cultivars included Chuandou-16 (CD-16, shade-intolerant plant) and Tianlong No. 1 (TL-1, shade-tolerant plant). The classical wide and narrow row planting was adopted in maize–soybean strip intercropping system (Yang et al., 2014; Cheng et al., 2020), with 2 m of each strip width and 6 m of each strip length (Figures 2A,C). Regardless of strip intercropping or monoculture, the planting density of TL-1 and CD-16 was 20 plants/m^2^, and the planting density of ZH-505 was 10 plants/m^2^. In the monocropping system, the same planting way as strip intercropping soybean without maize strip was conducted as the control (Figures 2B,D). The maize and soybean with few differences in growth period were directly broadcast in the field in the form of seeds. The area of each individual treatment plot was 12 m^2^ for both strip intercropping and monocropping, with a row × plant spacing of 40 cm × 20 cm for maize and a row × plant spacing of 40 cm × 10 cm for soybean. Each treatment that includes six biological repetitions was randomly planted in the whole experimental field. As base fertilizer for maize, 80 g/m^2^ of compound fertilizer (N: P: K = 15: 15: 15) was applied before sowing. The urea (*N* ≥ 46%) of 7.8 g/m^2^ and 13.2 g/m^2^ was applied at jointing stage and heading stage, respectively (Cheng et al., 2020), whereas soybean did not apply fertilizer in the whole growth period because of fertile experimental site.

### Sampling Time and Measurements

After 35 days of sowing, the samples of nine soybean plants with uniform and continuous growth for each treatment were collected every 14 days until harvest, with a total of five times. These samples were used for physiological and biochemical analyses. The collecting data information included the lodging rate (LR), LRI, PH, SD, LC, and activity of 4CL and PAL, canopy structure parameters that include LAI, MLA, and TC. Additionally, the Pn of the inverted trifoliate (PnIT, net photosynthetic rate), the average Pn of the whole plant (APnWP), and also stem anatomical structure that includes vascular, phloem, xylem, and pith were measured at R1 stage (early blooming stage). Grain yield and yield composition that include seed yield, full-pods, non-full-pods, and branches were measured at mature stage.

### Lodging Rate and Lodging Resistance Index

Lodging rate (Eq. 1) was calculated by randomly investigating a soybean strip without damage for each treatment according to the previous method (Cheng et al., 2020). LRI (Eq. 2) from the third to fifth internodes at the base of soybean stem, as an important and comprehensive stem strength index, was measured as reported earlier (Hussain et al., 2020a,b).


(1) $$LR\left(\%\right)=\frac{T\,N\,L\,S\,P}{T\,N\,S\,P}*100$$

where the LR represents the lodging rate of soybean in a plot, the TNLPP represents the total number of lodging soybeans in a plot, and the TNPP represents the total number of soybeans in a plot.


(2) $$L\,R\,I=\frac{S\,B\,R}{M\,S\,L*A\,G\,W}$$

where the LRI represents the lodging resistance index of soybean stem, SBR represents soybean stem bending resistance, the MSL represents the main stem length of soybean, and the AGW represents the above-ground biomass fresh weight of soybean.

### Leaf Area Index, Mean Leaf Angle, and Transmission Coefficient

The LAI, MLA, and TC of soybean canopy of each treatment were measured by Digital Plant Canopy Imager (CI-110, Zealquest Scientific Technology Co., Ltd., China), which measured four uniform measuring points in every treatment between two rows of soybean and took three more diagonal cells for repetition.

### Pn of the Inverted Trifoliate and Average Net Photosynthetic Rate of the Whole Plant

Between 9:00 a.m. to 11:00 a.m. on a sunny day, the PnIT and APnWP were measured with the portable photosynthetic analyzer LI-COR 6400 (Li-Cor Inc., Lincoln, NE, United States) at R1 stage of soybean. Totally, five plants with uniform growth were selected from each treatment and three repetitions, according to the earlier reported research (Cheng et al., 2020).

### Vascular, Phloem, Xylem, and Pith of Soybean

**FIGURE 3:**
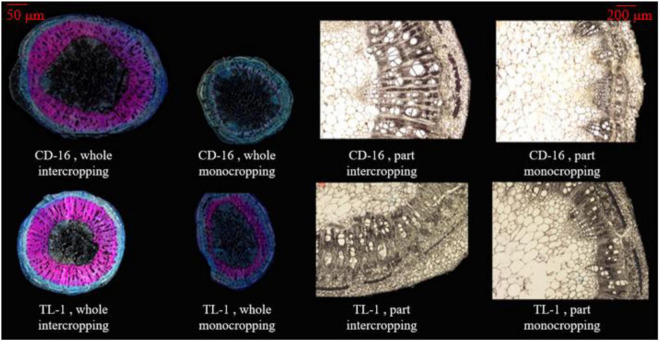
The anatomical structure of soybean stem, whole section **(left)**, and local section **(right)**. The xylem in the stem is dyed pink by turning safranin, using to characterize the LC. The phloem in the stem is dyed light blue by fixed o-fast green dye, using to characterize the cellulose content. In the local section, the white bundle structure is the vascular bundle.

The stem anatomical structure that includes vascular, phloem, xylem, and pith were measured according to the previous method (Rajput et al., 2018) with slight modification. The third internode of soybean stem was kept in FAA fixing solution for one month to soften. According to the following Table 1, the stem samples were dehydrated and transparent. Next, the stem samples were processed according to the following steps. ➀ Soaking wax: the paraffin wax (melting point: 57–63°C) was put into a beaker and melted it in a water bath pan (DXY-5H, Shenzhen Dingxinyi Experimental equipment Co., Ltd., China), in which the samples were soaked for about 8 h. ➁ Slicing: the wax block with stem samples was fixed on the slicer (RM 2245, Leica Microsystems Ltd., Wetzlar, Germany), and the slice thickness was set to 10 μm and sliced automatically (the blade was changed frequently). ➂ Exhibition: the cut samples were unfolded in a water bath pan (40°C), then placed on the slide glass (marked), and put the slide glass on the baking machine (HI 1210, Leica Microsystems Ltd., Germany) at 40°C for drying. ➃ Dewaxing: in a ventilated place, the slide glasses containing the samples were dissolved in a container containing xylene for 30 min and dried. ➄ Dyeing: the safranin was dropped on the stem samples for 10 min, slowly removed by distilling water, and dried naturally. Then, the fixed o-fast green dye was dropped on the treated samples for 10 s, slowly removed by distilling water, and dried naturally. ➅ Decolorization and observation: the slide glasses containing the samples were put in 75% alcohol to remove the residual dye and observed with a microscope (M205 FA, Leica Microsystems Ltd., Wetzlar Germany) after dried naturally. The final result is shown in Figure 3.

**TABLE 1:** Proportion of mixed chemical reagents and treatment time.

Mixed chemical reagent	Time
50% ethanol + 50% distilled water	6 h
50% ethanol + 30% *n*-butanol + 20 distilled water	5 h
40% ethanol + 50% *n*-butanol + 10% distilled water	4 h
30% ethanol + 80% *n*-butanol	3 h
10% ethanol + 90% *n*-butanol	2 h
100% *n*-butanol	1 h

### Seed Yield and Yield Composition

A total of 15 soybean plants with uniform and continuous growth were selected for each treatment. The number of full-pods, non-full-pods, and branches per plant, and also grain yield per unit area of soybean, were measured after natural drying. Likewise, 15 maize plants with uniform and continuous growth after natural drying were selected to measure grain yield per unit area.

### Plant Height, Stem Diameter, Lignin Content, and Activity of 4CL and Phenylalanine Ammonia Lyase

The stem length of soybean plant was regarded as PH, which was measured with flexible rule. The SD in the middle of the third internode of the stem was measured with the Vernier caliper. The LC, 4CL, and PAL from the third to fifth internode of the stem were determined by the test kits (Lignin, 4CL and PAL Test Kits, Suzhou Grace Biotechnology Co., Ltd., China) according to the previous methods (Cheng et al., 2020).

### The Shading-Tolerance Coefficient and Comprehensive Shading-Tolerance Coefficient

Shading-tolerance coefficient (Eq. 3) was used to describe the changes in target traits value of soybean between intercropping and monocropping, respectively. The CSTC (Eq. 4) was used to objectively evaluate the shade tolerance of soybean in intercropping systems (Li et al., 2014).


(3) $$S\,T\,C_{j}=1-\frac{\left|T\,T\,V_{i}-T\,T\,V_{m}\right|}{T\,T\,V_{m}}$$

where the STC represents shading-tolerance coefficient. The j represents different target traits of soybean. The TTV_*i*_ and TTV_*m*_ represent target traits value of soybean in monocropping and intercropping, respectively. When the value of STC is large, which means the target trait of soybean is less affected by shading; otherwise, it is more affected by shading.


(4) $$C\,S\,T\,C=\frac{1}{n}\,\sum_{j=1}^{n}S\,T\,C_{j}$$

where the CSTC represents CSTC. The n represents the number of target character of soybean. The j represents different target traits of soybean. When the value of CSTC is large, which means in the low-light environment, soybean shows a strong shade tolerance; otherwise, it possesses a weak shade tolerance (Liang and Li, 2004; Li et al., 2014).

### Statistical Analysis of Data and Graphing

All data were collected and stored by Microsoft Excel 2019 software. The Adobe Photoshop 2020 software was used to draw the maize and soybean plants and image typesetting. The Image Pro Plus software was used to process and calculate the areas of stem cross-section, vascular, phloem, xylem, and pith, etc. The LRI, LC, Pn, and the areas of stem cross-section, vascular, phloem, xylem, and pith and also seed yield, full-pods, non-full-pods, and branches were subjected to two-way ANOVA using the Origin Pro 2021 software. Significant differences among means of every treatment were separated according to Fisher’s LSD at the level of *p* ≤ 0.05. The *t*-test at *p* ≤ 0.05 was applied for correlation analysis. The LR, PH, SD LAI, MLA, and TC were subjected to one-way ANOVA to assess the effects of shading of maize and variety difference in this study.

## Results

### Seed Yield and Yield Composition

In the strip intercropping condition, the seed yield, the number of full-pods, and branches of CD-16 and TL-1 were significantly lower than those of monoculture, whereas the number of non-full-pods was the opposite (Table 2). The seed yield of TL-1 was 13.5% higher than that of CD-16 in the monocropping, whereas 21.8% higher than that of CD-16 in the strip intercropping (average of 2 years) (Table 2). A number of full-pods, non-full-pods, and branches of TL-1 were 16.6, 17.0, and 9.0% higher than those of CD-16, respectively, in the monocropping (Table 2). Similarly, in the strip intercropping, a number of full-pods, non-full-pods, and branches of TL-1 were 26.6, 22.4, and 29.6% higher than those of CD-16, respectively (Table 2). However, there was no significant difference between monoculture and intercropping in maize yield. The result showed that, compared with CD-16, the low decrease in grain yield of TL-1 in strip intercropping was due to its relatively high the number of full-pods and branches.

**TABLE 2:** The seed yield and composition of soybean and maize yield response to soybean cultivars and patterns in 2020 and 2021.

Years	Treatments		Soybean				Maize
			Seed yield	Full-pods	Non-full-pods	Branches	Seed yield
			g/m^2^	Numbers/plant	Numbers/plant	Numbers/plant	g/m^2^
2020	Inter.	CD-16	119.72^d^	23.00^d^	12.67^b^	1.33^d^	1248^a^
		TL-1	163.86^c^	31.33^c^	16.33^a^	1.89^c^	1260^a^
		*P*_*SV*_	**	**	**	**	n.s.
	Mono.	CD-16	288.21^b^	61.33^b^	7.67^d^	4.33^b^	1105^a^
		TL-1	323.86^a^	73.51^a^	9.24^c^	4.76^a^	
		*P*_*SV*_	*	**	**	**	–
	*P*_*PP*_		*	**	**	**	–
	*P*_SV*PP_		**	**	*	*	–
2021	Inter.	CD-16	98.45^d^	15.4^d^	–	–	1470^a^
		TL-1	115.32^c^	23.87^c^	–	–	1432^a^
		*P*_*SV*_	**	**	–	–	n.s.
	Mono.	CD-16	308.2^b^	75.26^b^	–	–	–
		TL-1	365.54^a^	88.83^a^	–	–	
		*P*_*SV*_	**	**	–	–	–
	*P*_*PP*_		**	**	–	–	–
	*P*_SV*PP_		**	**	–	–	–

Plants are grown in the strip intercropping (Inter.) and monocropping (Mono.). SV represented soybean variety. PP represents planting pattern. Data are average of three replicates. Different lowercase letters in the same column indicate significant differences among different cultivars (p < 0.05). p-Value is the result from two-way ANOVA. The * and ** indicate significant differences among different cultivars and planting patterns at the levels of p < 0.05 and p < 0.01, respectively. The n.s. means not significant at the level of p ≥ 0.05. The – indicates no data here.

### Lodging Rate and Lodging Resistance Index

**FIGURE 4:**
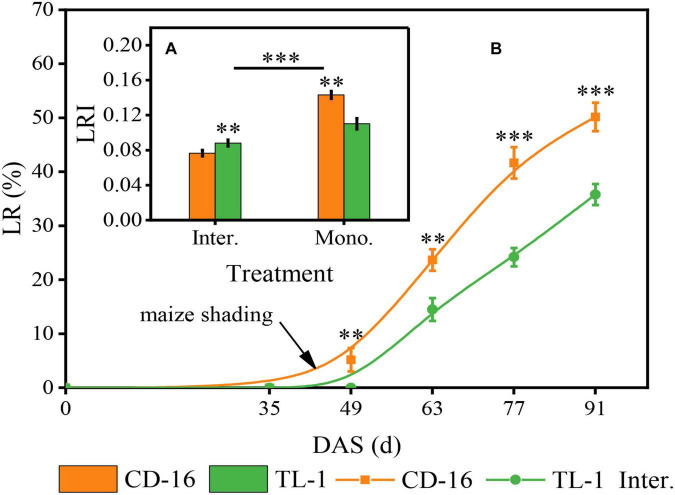
Effects of different cultivars and planting patterns on LR **(A)** and LRI **(B)**. The ^**^ and ^***^ indicate significant differences between CD-16 and TL-1 at the levels of *p* < 0.01 and *p* < 0.001, respectively. The *** on the black short line indicates extremely significant differences between intercropping and monocropping at the levels of *p* < 0.001. Data are average of three replicates. Error bars represent standard errors. Mono. and Inter. are the abbreviation of monoculture and strip intercropping, respectively. DAS represents the number of days after sowing.

Different soybean cultivars and planting patterns had significant impacts on LR (Figure 4A) and LRI (Figure 4B) of soybean. We noticed that whether CD-16 or TL-1, the lodging only occurred in the strip intercropping system, and with the development of soybean and maize growth, the LR increased continuously (Figure 4B). However, the LR of TL-1 was significantly lower than that of CD-16 (38.2% lower on average), and the lodging time of TL-1 in the field was approximately 14 days later than that of CD-16 (Figure 4B). To explain these phenomena, we further measured the LRI of soybean and found that the LRI of soybean in the strip intercropping was significantly lower than that of monocropping regardless of CD-16 or TL-1 (Figure 4A). Furthermore, the LRI of TL-1 was 30.0% lower than that of CD-16 in monocropping; however, but it was 15.1% higher than that of CD-16 in the strip intercropping (Figure 4A). This phenomenon could be explained according to the Eq. 2. Consequently, in the maize–soybean strip intercropping system, TL-1, a shade-tolerant plant, had relatively strong stem strength compared with CD-16, which was beneficial to maintain the upright growth of the plant.

### Canopy Structure of Soybean

**FIGURE 5:**
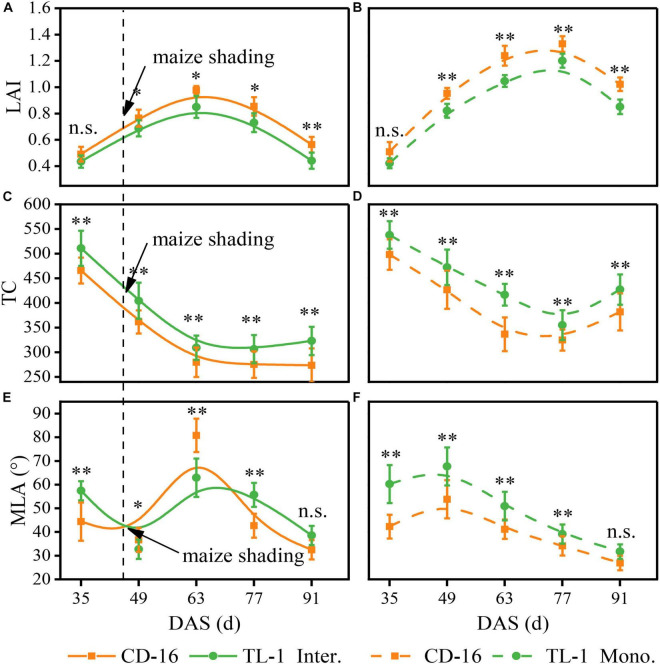
Effects of different cultivars and planting patterns on LAI **(A,B)**, TC **(C,D)** and MLA **(E,F)**. The * and ^**^ indicate significant differences between CD-16 and TL-1 at the levels of *P* < 0.05 and *P* < 0.01, respectively. The n.s. means not significant at the level of *p* ≥ 0.05. The vertical black dotted line indicates that the maize begins to shade the intercropping soybean at this time. Data are average of three replicates. Error bars represent standard errors. The solid line indicates strip intercropping, and the dashed line indicates monoculture. The Mono. and Inter. are the abbreviation of monoculture and strip intercropping, respectively. DAS represents the number of days after sowing.

There was no significant difference in the LAI between TL-1 and CD-16 before soybean shaded by maize (0–49 days after sowing) in the strip intercropping (Figures 5A,B). Thereafter, the LAI of TL-1 was significantly lower than that of CD-16 during the whole later growth period of soybean, irrespective of the cropping system (Figures 5A,B). Notably, the LAI of TL-1 in the monocropping was 20.01% lower than that of CD-16, whereas that of TL-1 in the strip intercropping was only 16.0% lower than that of CD-16 (average of the last four sampling times). Additionally, the peak time of LAI of TL-1 and CD-16 in strip intercropping (63 days after sowing) was approximately 14 days earlier than that in monoculture (77 days after sowing), due to the shading stress of maize on soybean (Figures 5A,B).

Irrespective of the cropping system, the TC of TL-1 was significantly higher than that of CD-16 during the whole growth period of soybean (Figures 5C,D). After shaded soybean by maize, the TC of TL-1 and CD-16 in the strip intercropping was lower than that of in monocropping (Figures 5C,D). It was noteworthy that the TC of TL-1 in the monocropping was 10.91% higher than that of CD-16, but the TC of TL-1 was 11.42% higher than that of CD-16 (average of the last four sampling times) in the late maize shading.

Whether it was in strip intercropping or monoculture, the MLA of TL-1 was significantly higher than that of CD-16 from 0 to 49 days after sowing, whereas that of TL-1 in the strip intercropping was lower than that of CD-16 from 49 to 63 days after sowing (Figures 5E,F). Immediately, the MLA of T-1L and CD-16 decreased rapidly in the strip intercropping after the 63rd day of sowing, but the decreasing rate of TL-1 was faster as compared to CD-16 (Figures 5E,F). What should be of concern was that the MLA of TL-1 in the monocropping was 20.55% lower than that of CD-16, whereas the MLA of TL-1 was only 4.15% lower than that of CD-16 (average of the last four sampling times) in the late maize shading.

In a word, the shading of maize decreased the LAI and TC, but increased the MLA of intercropping soybean canopy, regardless of CD-16 or TL-1; however, the variation range of these canopy parameters of TL-1 were lower as compared to CD-16. The result showed that in the strip intercropping system, the canopy spatial structure of shade-tolerant plants (TL-1) was slightly changed by the shading stress, which was conducive to increasing light energy capture and the photosynthesis of leaves at different canopy levels in a low-light environment.

### Photosynthetic Efficiency

**FIGURE 6:**
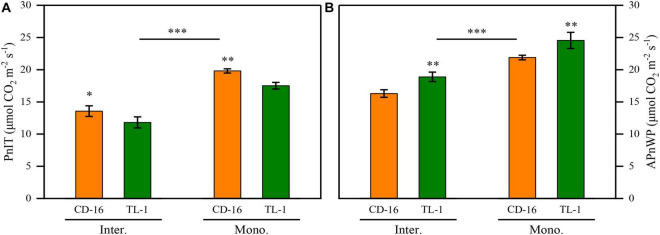
Effects of different cultivars and planting patterns on the PnIT **(A)** and the APnWP **(B)** at R1 stage. The * and ^**^ indicate significant differences between CD-16 and TL-1 at the levels of *p* < 0.05 and *p* < 0.01, respectively. The ^***^ on the black short line indicates extremely significant differences between intercropping and monocropping at the levels of *p* < 0.001. Data are average of three replicates. Error bars represent standard errors. Mono. and Inter. are the abbreviation of monoculture and strip intercropping, respectively.

Regardless of CD-16 or TL-1, the PnIT and APnWP of strip intercropping soybean were significantly lower than those of monoculture soybean at R1 stage (Figures 6A,B). Furthermore, the PnIT of TL-1 was obviously lower than that of CD-16 (14.0% lower on average), irrespective of planting patterns (Figure 6A). However, compared with CD-16, most of the leaves of TL-1 in both strip intercropping and monocropping maintained a relatively high level of Pn, which led to a higher APnWP of TL-1 (12.3% higher on average) (Figure 6B). The result showed that in the strip intercropping system, the shade-tolerant plant (TL-1) which was not easy to change in spatial structure could maintain the relatively high Pn of leaves at different canopy levels.

### Morphology and Anatomical Structure of Stem

**FIGURE 7:**
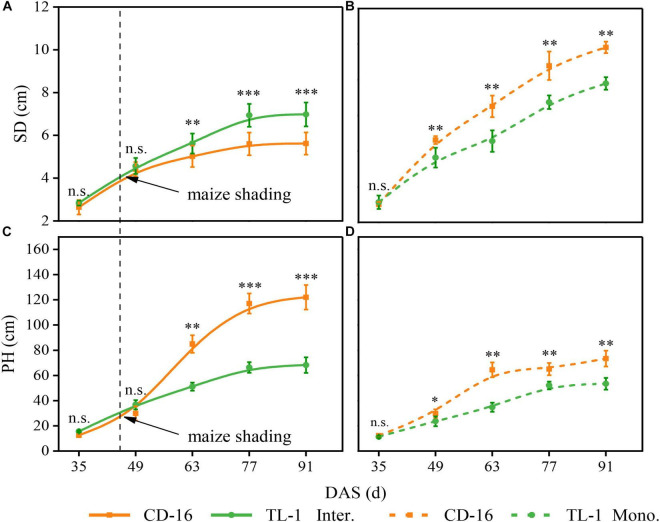
Effects of different cultivars and planting patterns on SD **(A,B)** and PH **(C,D)**. The *, ^**^, and ^***^ indicate significant differences between CD-16 and TL-1 at the levels of *p* < 0.05, *p* < 0.01, and *p* < 0.001, respectively. The n.s. means not significant at the level of *p* ≥ 0.05. The vertical black dotted line indicates that the maize begins to shade the intercropping soybean at this time. Data are average of three replicates. Error bars represent standard errors. The solid line indicates strip intercropping, and the dashed line indicates monoculture. Mono. and Inter. are the abbreviation of monoculture and strip intercropping, respectively. DAS represents the number of days after sowing.

Compared with monoculture, maize shading reduced the SD but increased the PH of strip intercropped soybean regardless of CD-16 or TL-1, but the change range of SD and PH of TL-1 was smaller than that of CD-16 (Figures 7A–D). However, the SD of TL-1 in the strip intercropping was even higher than that of CD-16 as compared to monocropping (Figure 7A).

Anatomical structure of stems showed that planting patterns, soybean cultivars, and their interactions have significant effects on the number of vascular bundles and the area ratio of vascular/stem, phloem/stem, xylem/stem, and pith/stem at R1 stage (Table 3 and Figure 3). The number of vascular bundles and the area ratio of phloem/stem and xylem/stem of TL-1 in the monocropping were significantly lower than those of CD-16 (Table 3 and Figure 3). In addition, for TL-1, the percentage of decline in the number of vascular bundles between monoculture and intercropping was 4.08%, whereas for CD-16, the percentage of decline was 19.50% (Table 3). In contrast, those anatomical structure parameters of TL-1 in the strip intercropping were significantly higher than CD-16 (Table 3 and Figure 3).

**TABLE 3:** The number of vascular bundles and the area ratio of vascular/stem, phloem/stem, xylem/stem, and pith/stem response to planting patterns and soybean cultivars at R1 stage.

Treatment		Vascular	Vascular/stem	Phloem/stem	Xylem/stem	Pith/stem
		Unit/mm^2^	%	%	%	%
Mono.	CD-16	57.00^a^	0.96^c^	17.17^b^	41.49^a^	40.35^c^
	TL-1	49.00^b^	1.28^b^	15.40^c^	22.64^d^	43.72^a^
	*P*_*SV*_	*	*	*	**	*
Inter.	CD-16	25.00^d^	0.90^c^	18.73^b^	30.23^c^	41.96^b^
	TL-1	47.00^c^	1.59^a^	42.71^a^	36.80^b^	42.71^ab^
*P*_*SV*_		**	*	*	**	**
*P*_*PP*_		**	**	**	**	**
*P*_SV*PP_		**	*	**	**	**

Plants are grown in the strip intercropping (Inter.) and monocropping (Mono.). SV represented soybean variety. PP represents planting pattern. Data are average of three replicates. Different lowercase letters in the same column indicate significant differences among different cultivars (p < 0.05). p-Value is the result from two-way ANOVA. The * and ** indicate significant differences among different cultivars and planting patterns at the levels of p < 0.05 and p < 0.01, respectively. The – indicates no data here.

Consequently, in the maize–soybean strip intercropping system, there were relatively high number of vascular bundles and the area of vascular, phloem, xylem, and pith in shade-tolerant plant (TL-1) compared with CD-16, which was beneficial to maintain a relatively thick stem.

### Lignin Content and Related Enzyme Activity

**FIGURE 8:**
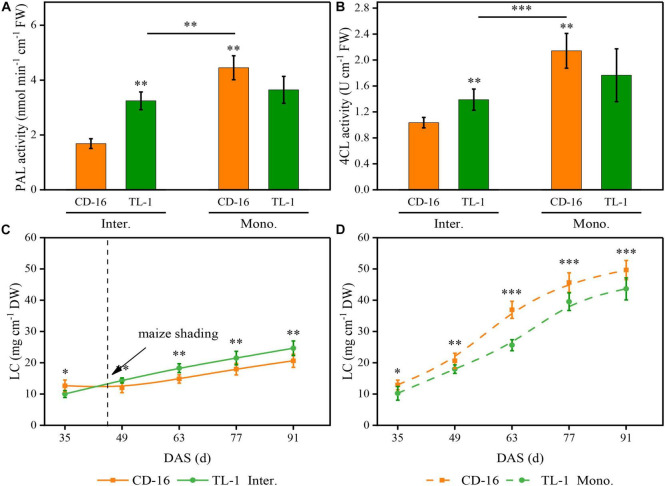
Effects of different cultivars and planting patterns on PAL **(A)**, 4CL **(B)**, and LC **(C,D)**. The *, ^**^, and ^***^ Proportion of mixed chemical reagents indicate significant differences between CD-16 and TL-1 at the levels of *p* < 0.05, *p* < 0.01, and *p* < 0.001, respectively. The ^**^ and ^***^ on the black short line indicate significant differences between intercropping and monocropping at the levels of *p* < 0.005 and *p* < 0.001, respectively. The vertical black dotted line indicates that the maize begins to shade the intercropping soybean at this time. Data are average of three replicates. Error bars represent standard errors. The solid line and dashed line indicate strip intercropping and monoculture, respectively. Mono. and Inter. are the abbreviation of monoculture and strip intercropping, respectively. DAS represents the number of days after sowing.

As compared to monoculture, regardless of CD-16 or TL-1, maize shading decreased the activity of PAL (Figure 8A) and 4CL (Figure 8B) of strip intercropped soybean, which led to the LC of intercropping soybean decreasing (Figure 8C). Furthermore, compared with CD-16, the TL-1 had lower PAL and 4CL activities and lower LC in monoculture (lower than 22.1, 21.4, and 21.0%, respectively), but it was opposite in the strip intercropping (higher than 116.4, 30.6, and 12.0%, respectively) (Figures 8A–D). Consequently, in the maize–soybean strip intercropping system, there was a relatively high activity of PAL and 4CL in shade-tolerant plant (TL-1) compared with CD-16, which was beneficial to maintain a relatively high LC of stem.

### Correlation Analysis

**FIGURE 9:**
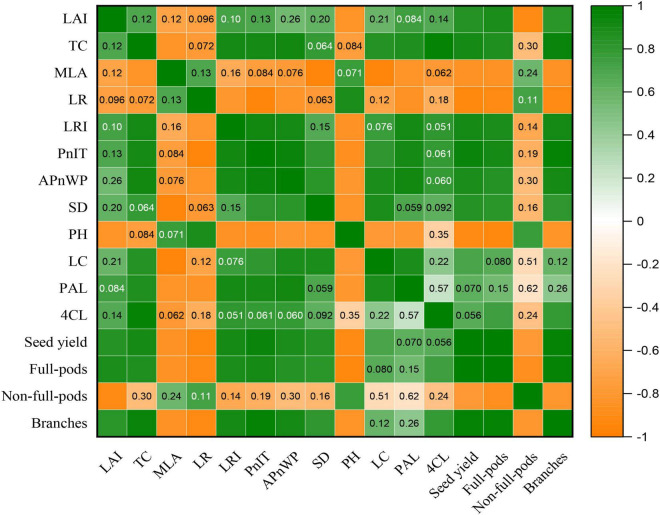
Correlation analysis of LAI, TC, MLA, LR, LRI, the PnIT, the APnWP, SD, PH, LC, PAL, 4CL, seed yield, full-pods, non-full-pods, and branches by using the *t*-test at *p* ≤ 0.05 for both TL-1 and CD-16 in the strip intercropping. Orange means positive correlation, and green means negative correlation. The darkness of the color represents strength of correlation. The numbers in the boxes indicate *p*-value, and a box without numbers indicates a strong correlation.

To demonstrate the effect of plant canopy structure on stem morphology and yield components of soybean, we further analyzed the correlation between the canopy structure of plants and the key morphological and physiological parameters of stem and also yield, etc. (Figure 9). The result showed that the LAI was significantly and positively correlated with seed yield, full-pods, and branches, but significantly and negatively correlated with PH and non-full-pods (Figure 9). Additionally, the TC significantly and positively correlated with LRI, PnIT, APnWP, LC, PAL, 4CL, seed yield, full-pods, and branches, whereas significant negative correlation existed between the TC and MLA (Figure 9). Additionally, there was significant and negative correlation between the MLA and SD, LC, PAL, seed yield, full-pods, and branches (Figure 9). Consequently, these parameters, such as LAI, TC, and MLA, could be potentially used to evaluate canopy structure of plants and reflect the growth status of plants and final yield formation.

### Evaluation of Shading-Tolerance Target Traits in Soybean

**FIGURE 10:**
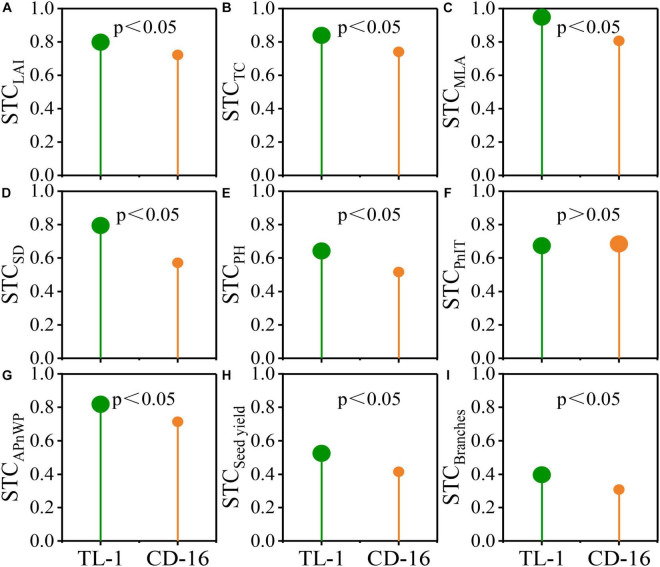
Evaluation of shade-tolerance target traits such as LAI **(A)**, TC **(B)**, MLA **(C)**, SD **(D)**, PH **(E)**, the PnIT **(F)**, the APnWP, **(G)**, seed yield **(H)**, and branches **(I)** in soybean at the levels of *p* ≤ 0.05.

The STC of shading-tolerance target traits in TL-1 (e.g., LAI, MLA, SD, PH, APnWP, seed yield, and branches) significantly larger than those of CD-16 (Figures 10A–E,G–I). The STC of PnIT was not significant in TL-1 and CD-16 (Figure 10F). The possible reason is that the decrease of PnIT caused by shading stress is roughly the same, regardless of CD-16 or TL-1. Moreover, compared with CD-16 (CSTC = 0.608), TL-1 (CSTC = 0.716) had a higher CSTC, which indicated that it had a stronger shade tolerance in strip intercropping system.

## Discussion

### Photosynthesis and Yield in Strip Intercropping Enhanced by Regulating the Canopy Structure

In the maize–soybean strip intercropping system, soybean was vulnerable to shading stress from maize during the intergrowth, which thus affected its canopy structure and photosynthesis (Hideki et al., 2005; Feng et al., 2019). A large number of studies have shown that crop canopy structure was closely related to interception and utilization of photosynthetic active radiation (Yang et al., 2014; Feng et al., 2016; Charbonnier et al., 2017). In this study, the LAI, MLA, and TC of strip intercropped soybean were significantly lower than those of monoculture (Figure 5). Consequently, a serious impact was that the PnIT and APnWP of strip intercropped soybean were significantly lower than those of monoculture soybean at R1 stage (Figures 6A,B). In addition, the photosynthetic rate of intercropped soybean had been proved to be weaker than that of monoculture soybean (Su et al., 2014; Yao et al., 2017; Fan et al., 2018; Raza et al., 2019), which provided additional evidence to support that the intercropped soybean with lower the PnIT and APnWP experienced a shaded environment. Another important evidence showed that the low-light intensity aggravated senescence of leaves, which results in a decrease in leaf numbers (Lim et al., 2007; Zhou et al., 2019a,b), which in turn weakened canopy structure and photosynthesis of strip intercropped soybean (Figures 5, 6).

Variations in leaf area distributions of different soybean cultivars in the field grown were largely determined by differences in the light intensities at the lower canopy levels, but independent of the angle of leaf inclination (Blad and Baker, 1972). In this study, the shade-tolerant soybean TL-1 had high STC of canopy structure (STC_LAI_ = 0.799, STC_TC_ = 0.839, and STC_MLA_ = 0.949) (Figures 10A–C), which was conducive to maintaining the relative stability of spatial structure of the canopy in low-light environment. Importantly, compared with the shade-intolerant soybean CD-16, the shade-tolerant soybean TL-1 had a more perfect canopy structure such as higher TC and lower LAI and MLA (Figure 5), which would allow greater penetration of light to the lower canopy levels (Blad and Baker, 1972; Sakowska et al., 2018). Therefore, compared with shade-intolerant soybean CD-16, the shade-tolerant soybean TL-1 had a higher APnWP (Figure 6B), even though its PnIT was lower (Figure 6A). As a result, a good canopy structure of soybean is the prerequisite for maintaining high APnWP in low-radiation areas. However, in the intercropping system, the molecular mechanism of low-light environment regulating soybean canopy structure (LAI, TC, and MLA) was still unclear, which needs further research.

Most crops in the intercropping system showed yield advantages as compared to the corresponding monoculture (Li et al., 2001, 2020a; Ghosh, 2004; Chen et al., 2019; Feng et al., 2019). In this study, the soybeans in the strip intercropping system showed no yield advantages as compared to monoculture, irrespective of cultivars, across the two years in field experiments (Table 2). One possible explanation was that the canopy morphological formation of strip intercropped soybean was severely inhibited during the intergrowth with maize, which results in plant lodging (Figure 4B) and yield reduction (Table 2). Correlation analysis showed that the canopy structure of strip intercropped soybean was significantly correlated with LR and yield (Figure 9). A compact-type canopy structure was beneficial to intercept more light energy to promote stem morphogenesis and yield formation (Wang et al., 2017; Li et al., 2020b). In addition, the low dry matter allocation index limiting the yield of strip intercropped soybean might be another reason, because in the reproductive stage, the dense canopy structure with relatively low PAR reduced the number of full-pods and branches (Table 2), which increases the allocation of dry matter to leaves and stems and thus decreases the allocation of photosynthetic products to seeds (Board, 2000; Liu et al., 2010; Cheng et al., 2020). In contrast, the shade-tolerant soybean TL-1 with high STC (STC_Seed yield_ = 0.526 and STC_Branches_ = 0.397) (Figures 10H,I) had relatively high seed yield, full-pods, and branches in the strip intercropping system (Table 2). This was one of the important strategies to increase the yield of shade-tolerant soybean in areas with low-light radiation.

### Stem Morphological and Physiological Responses to Canopy Structure

Crops with the perfect canopy structure were propitious to intercept more light energy to promote stem morphogenesis and yield formation (Board, 2000; Wang et al., 2017; Cheng et al., 2020; Li et al., 2020b), especially in the condition of intercropping. Some studies have shown that crops in intercropping showed strong shade avoidance responses, which include thinner stem thickness, longer stem, and petiole and also lower PAR inside the canopy and photosynthetic capacity (Yang et al., 2014; Liu et al., 2016; Fan et al., 2018). In this study, regardless of shade-intolerant soybean CD-16 or shade-tolerant soybean TL-1, The stem of strip intercropped soybean was obviously weaker than that of monoculture in terms of external morphology (lower SD and higher PH) (Figure 7) or internal structure (less vascular bundles and smaller area ratio of vascular/stem, phloem/stem, xylem/stem, and pith/stem) (Table 3 and Figure 6), which provided additional evidence to support that there was less PAR inside the canopy of intercropped soybean. In addition, regardless of soybean cultivars, some studies (Cheng et al., 2020; Hussain et al., 2021) were consistent with the observations that the strip intercropped soybean with an unfilled canopy showed a lower activity of PAL and 4CL (Figures 8A,B), and also LC (Figures 8C,D) compared with monocropping. Regardless of planting system or cultivars, the stem with low LC was one of the key factors for plant lodging and yield reduction (Lourenco et al., 2016; Hussain et al., 2020a; Figures 4, 8C,D). Additionally, the cellulose content in soybean stem was also closely related to plant lodging in intercropping (Liu et al., 2016). In this study, the canopy structure, LC, stem morphology, leaf photosynthesis, and yield components were also closely correlated (Figure 9). Thus, it could be seen that improving the canopy structure and the LC in the stem during intercropping was beneficial to decrease the lodging risk of soybean.

Fortunately, soybean cultivars of different genotypes had different responses to various shade environments. The shade-tolerant soybean (CSTC = 0.713) slightly changed its spatial structure of canopy and stem morphology and physiology in maize–soybean strip intercropping system (Figures 3, 5, 7). Some studies showed the morphology of shade-tolerant plants had lower plasticity (Gong et al., 2015; Chmura et al., 2017), so as to maintain the canopy structure stability of the plant in low-light conditions. Therefore, compared to the shade-intolerant CD-16, the shade-tolerant TL-1 with a relatively perfect canopy had the stronger external morphology (Figure 7) and internal structure (Table 3 and Figure 3) in the strip intercropping, in favor of increasing LRI and reducing the LR of soybean in the field (Figure 4). Interestingly, for TL-1, the percentage of decline in the number of vascular bundles between monoculture and intercropping was 4.08%, whereas for CD-16, the percentage of decline was 19.50% (Table 3), which shows that the TL-1 could slightly reduce the number of vascular bundles in the stem and maintain the stem strength in strip intercropping (Hussain et al., 2021). Besides, high-light intensity was one of the important abiotic factors to improve photosynthesis of plants (Yang et al., 2014; Xue et al., 2016; Feng et al., 2019; Zhou et al., 2019a,b). As compared to shade-intolerant CD-16, most of the leaves of shade-tolerant TL-1 in both strip intercropping and monocropping maintained a relatively high level of APnWP (Figure 6B), which led to a higher accumulation of photosynthetic outcome in stem (Figures 3, 7, 8 and Table 3) and grains (Table 2). Finally, intercropping could improve the microclimate conditions of coffee plants, such as lowering temperature and irradiance level and increasing air relative humidity (Partelli et al., 2014). Thus, what is the relationship between soybean field microclimate and plant lodging resistance and yield formation in the maize–soybean strip intercropping systems? Which needs to be further explored.

## Conclusion

Our results demonstrated that compared with shade-intolerant soybean, the shade-tolerant soybean (CSTC = 0.713) slightly changed its spatial structure of canopy and stem morphology and physiology in maize–soybean strip intercropping system, especially in the middle and later growth stages. On the one hand, the canopy structure of shade-tolerant soybean showed relatively high TC and relatively low LAI and MLA. Importantly, the shade-tolerant soybean had high STC of canopy structure (STC_LAI_ = 0.799, STC_TC_ = 0.839, and STC_MLA_ = 0.949), which was conducive to maintaining the relative stability of spatial structure of the canopy in low-light environment. On the other hand, the stem of shade-tolerant soybean was obviously stronger than that of shade-intolerant plant in terms of external morphology (shorter PH and thicker stem), internal structure (more vascular bundles and higher area ratio of vascular/stem, phloem/stem, xylem/stem, and pith/stem), and physiological characteristics (higher LC and enzyme activity of PAL and 4CL). In brief, shade-tolerant soybean increased light energy capture and the photosynthesis of the different canopy levels to promote the morphological and physiological development of the stem and ultimately reduce the yield loss of the strip intercropping system. This study provided new insights into the mechanism of soybean lodging resistance in the strip intercropping system, which was of great significance for understanding the potential mechanism of how shade-tolerant plants reduce soybean yield loss in the strip intercropping system by regulating its spatial structure and the morphology and physiology of stems, especially in areas with low solar radiation. Moreover, the spatial structure of canopy with high TC and low LAI and MLA could be used as one of the important bases for screening soybean cultivars with shade resistance and lodging resistance in the future, especially in low radiation areas. However, the molecular mechanism of low-light regulating soybean canopy structure (LAI, TC, and MLA) needs further study, to provide theoretical guidance for cultivating plants with ideal canopy shape that can adapt to changing light environment in intercropping system.

## Data Availability Statement

The original contributions presented in the study are included in the article/supplementary material, further inquiries can be directed to the corresponding author/s.

## Author Contributions

BC and LW carried out the writing–riginal draft, data analysis and plotting. SZJ, RL, WBW, RY, TZ, and SJJ provided the manuscript overall guidance and some references. BC, LW, IA, and AR carried out the soybean sowing, investigation and analysis of physiological and biochemical. MX, CL, WY, and LY carried out the data curation. WL and WY provided financial support. All authors contributed to the article and approved the submitted version.

## Conflict of Interest

BC, RL, and SJJ was employed by the company Chengdu Da Mei Seeds Co., Ltd. The remaining authors declare that the research was conducted in the absence of any commercial or financial relationships that could be construed as a potential conflict of interest.

## Publisher’s Note

All claims expressed in this article are solely those of the authors and do not necessarily represent those of their affiliated organizations, or those of the publisher, the editors and the reviewers. Any product that may be evaluated in this article, or claim that may be made by its manufacturer, is not guaranteed or endorsed by the publisher.
